# Hypoxic signature of microRNAs in glioblastoma: insights from small RNA deep sequencing

**DOI:** 10.1186/1471-2164-15-686

**Published:** 2014-08-17

**Authors:** Rahul Agrawal, Priyatama Pandey, Prerana Jha, Vivek Dwivedi, Chitra Sarkar, Ritu Kulshreshtha

**Affiliations:** Department of Biochemical Engineering and Biotechnology, Indian Institute of Technology, 110016 New Delhi, India; School of Computational and Integrative sciences, Jawaharlal Nehru University, 110067 New Delhi, India; Department of Pathology, All India Institute of Medical Sciences, 110029 New Delhi, India

**Keywords:** Glioblastoma, MicroRNA, Deep sequencing, U87MG, U251MG, A172, miR-210

## Abstract

**Background:**

Hypoxia is a critical aspect of the glioma microenvironment and has been associated with poor prognosis and resistance to various therapies. However, the mechanisms responsible for hypoxic survival of glioma cells remain unclear. Recent studies strongly suggest that microRNAs act as critical mediators of the hypoxic response. We thus hypothesized their prominent role in hypoxia resistance in glioblastoma (GBM) and aimed to identify those.

**Results:**

With this study, we present the first detailed analysis of small RNA transcriptome of cell line U87MG, a grade IV glioma cell line, and its alteration under hypoxic condition. Based on deep sequencing and microarray data, we identify a set of hypoxia regulated microRNAs, with the miR-210-3p and its isomiRs showing highest induction in GBM cell lines U87MG and U251MG. We show miR-210-3p, miR-1275, miR-376c-3p, miR-23b-3p, miR-193a-3p and miR-145-5p to be up-regulated, while miR-92b-3p, miR-20a-5p, miR-10b-5p, miR-181a-2-3p and miR-185-5p are down-regulated by hypoxia. Interestingly, certain hypoxia-induced miRNAs are also known to be over-expressed in GBM tumors, suggesting that hypoxia may be one of the factors involved in establishing the miRNA signature of GBM. Transcription factor binding sites for Hypoxia inducible factor 1 A (HIF1A) were identified in the promoter region (5 kb upstream) of 30 hypoxia-induced miRNAs. HIF-1A over-expression and silencing studies show regulation of specific miRNAs, including miR-210-3p, to be HIF1A dependent. On the other hand, miR-210-3p leads to an increase in transcriptional activity of HIF and its target genes vascular endothelial growth factor (VEGF) and carbonic anhydrase 9 (CA9). MiR-210-3p levels were found to be high in GBM patient samples and showed good correlation with the known hypoxia markers CA9 and VEGF. We show that miR-210-3p promotes hypoxic survival and chemoresistance in GBM cells and targets a negative regulator of hypoxic response, HIF3A. Additionally, a total of 139 novel miRNAs were discovered by the analysis of deep sequencing data and three of these were found to be differentially expressed under hypoxia.

**Conclusions:**

Overall, our study reveals a novel miRNA signature of hypoxia in GBM and suggests miR-210-3p to be an oncogenic player and a novel potential intrinsic marker of hypoxia in glioblastoma.

**Electronic supplementary material:**

The online version of this article (doi:10.1186/1471-2164-15-686) contains supplementary material, which is available to authorized users.

## Background

Hypoxia, or low oxygenation, has emerged as an important factor in tumor biology and response to cancer treatment [1]. It has been shown to be a negative prognostic factor for several cancers, including those of the cervix, head and neck, prostate, pancreas, and brain [2]. GBM is the most common primary brain tumor, representing about 17% of all primary brain tumors and about 60-75% of all astrocytomas [3, 4]. The severity of tumor hypoxia is known to strongly correlate to tumor progression, metastasis, invasion, therapeutic resistance [5, 6]. High levels of hypoxic markers like VEGF, CA9 and OPN are correlated with poor prognosis for GBM patients [7, 8]. Recent report shows that the hypoxic microenvironment maintains glioblastoma stem cells and promotes reprogramming towards a cancer stem cell phenotype [9]. This suggests to target hypoxia, or hypoxia-regulated genes for therapeutic purposes. However, the results have been unsatisfactory so far, emphasizing the need to identify novel players in hypoxia signalling as potential targets for cancer therapy.

MicroRNAs (a class of small non-coding RNAs) have emerged as key players in cellular transformation and tumorigenesis and show great potential for cancer diagnostics and therapeutics [10, 11]. Recent studies strongly indicate that miRNAs are involved in the pathogenesis of GBM [12]. The first report demonstrating altered expression of microRNAs in GBM was published in 2005 [13]. MiR-21 was the first miRNA found strongly upregulated in six cell lines of GBM and is now established as an important oncogene that targets multiple components of p53 and transforming growth factor-beta (TGF-beta) pathways in GBM cells [14]. Functional analysis of other miRNAs aberrantly expressed in GBM, like miR-221/222, -34a, -146b and -10b, showed an influence on cell cycle, glioma cell migration and invasion and stem cell properties [15–17]. Recently, Moore *et al*. showed that during progression from low grade to high grade glioma, the amount of mature miRNAs increases in comparison to precursor hairpins [18].

Importantly, miRNAs have also been recently reported to act as critical mediators of hypoxia signalling [19]. The pioneering work by Ivan’s group shows that a specific set of hypoxia-regulated miRNAs (HRMs) modulates cell cycle, apoptosis and DNA repair pathways in response to hypoxia in breast cancer [20–22]. Since then, several studies have found that HRMs fine- tune their hypoxic response through cellular processes such as angiogenesis, cell cycle regulation, metabolism, apoptosis, metastasis, proliferation and resistance to anticancer therapy [23].

Considering the wide impact of miRNAs in hypoxic tumor biology, it seems important to identify and functionally characterize HRMs in GBM. This can give insight into the molecular mechanism of hypoxia-resistance in GBM and might have implication for GBM diagnosis and therapy. In this study, we used deep-sequencing profiling of small RNAs (sRNAs), along with microarray hybridization to study the expression pattern of miRNAs in response to hypoxia in a GBM cell line (U87MG). The advantage of deep sequencing over other detection methods, like northern blot analysis, RNAase protection assays, microarray chip techniques or real-time PCR, lies in the ability to detect low abundant and novel transcripts. The signature we obtained contains known as well as novel miRNAs. Subsets of these were found to carry HIF1A response elements (HREs) in their promoters and were shown to be HIF-1A regulated. Interestingly, a well-known HRM, miR-210-3p, was found to be highly induced in hypoxic glioma cell lines (U87MG and U251MG) as well as in hypoxic GBM tumor samples, suggesting its use as a hypoxia marker or therapeutic target in GBM. Based on our results, miR-210-3p promotes survival of GBM cells in the tumor microenvironment, promotes aggressiveness by imparting temozolomide resistance and targets HIF3A, which is known to function as a negative regulator of hypoxia-inducible gene expression [24].

## Methods

### Cell culture

Cell line U87MG was obtained from the National Centre for Cell Sciences, Pune. U251MG and A172 were kind gifts from Dr. Kunzang Chosdol (AIIMS, Delhi, India) and Dr. Ellora Sen (NBRC, Manesar, India), respectively. All cell lines were maintained in DMEM medium. The medium was supplemented with 10% Fetal Bovine Serum, 100 U/ml penicillin and 100 μg/ml streptomycin. It was incubated at 37°C and 5% CO_2_. To create hypoxic conditions, cells were grown in a hypoxia workstation (*In vivo* 200, Ruskinn Technology Ltd., UK) at 0.2% O_2_, 5% CO_2_ and 37°C.

### Glioblastoma patient samples

Thirty samples of human glioblastoma (GBM: WHO Grade IV) were obtained from the Neuropathology Laboratory of the Department of Pathology, All India Institute of Medical Sciences (AIIMS), New Delhi. The study was approved by the ethical committee of the institute. Cases were selected on the basis of availability of adequate tumor tissue. The haematoxylin- and eosin- (H&E) stained slides of these cases were reviewed and a concordant agreement was established on the diagnosis between three trained pathologists, based on the WHO 2007 tumor classification [25]. Clinical data, viz. age, sex and history were noted. The age range of the patients was from 19 to 80 years, and the mean age was about 51. The male to female ratio was around 1.5. All cases were *de novo* primary GBMs. Five control brain tissues were also obtained from cases of epilepsy surgery. We used the epileptic tissue as non-neoplastic reference, as this serves as best control. We avoided the use of tissue adjacent to the tumor as control, since there is the possibility of tumor cell infiltration.

### Transient and stable transfections

U87MG cells were seeded in 6 well plates (3×10^5^ cells/well) 24 h before transfection. Cells were transfected with plasmid (2.5 μg), using transfection reagent Lipofectamine 2000 (Invitrogen). In case of the miR-210 inhibitor and the respective control (Sigma), 30 nM of each were transfected. The plasmids – pCDNA3.1, pCDNA3.1-HIF1A, pLK0.1-shGFP and pLK0.1-shHIF1A were a kind gift from Dr. Mircea Ivan (Indiana University, IN).

### Construction of stable polyclonal cell lines

The miR-210 overexpressing vector (pBABE-miR-210) or the empty parent vector pBABE-puro were prepared as described in Crosby et al., [26]. The plasmids were then transfected into U87MG cells, and stable polyclonal cell lines were selected with 2 μg/ml puromycin for up to one month.

### Hypoxia, serum starvation and drug treatment

The stable polyclonal cell lines were seeded in 24-well plate (5 × 10^4^ cells/well) in triplicate. For hypoxic stress, the cells were placed 24 h post-seeding in a hypoxia chamber maintaining 0.2% oxygen and 5% CO2. For serum starvation, the supplemented medium was replaced with medium lacking serum. For drug treatment, temozolomide was added to a concentration of 5 μM. Cell survival was quantified at different time points using 3-(4,5-dimethylthiazole-2-yl)-2,5-diphenyl tetrazolium bromide (MTT) at 595 nm.

### RNA isolation

Cells were suspended in TRIzol (Invitrogen), and total RNA was isolated according to the manufacturer's protocol using the GeneJET™ RNA purification kit. To isolate RNA from tumor tissues, tissues were pre-treated with xylene and protease, followed by RNA isolation using an RNA isolation kit (Ambion) according to the manufacturer’s protocol. The concentration and purity of the extracted RNA was measured with a NANODROP 2000c spectrophotometer (Thermo Scientific). RNA was stored at -80°C.

### Small RNA preparation

Total RNA was isolated using TRIzol, and isolated RNA was run on a denaturing polyacrylamide gel. A section of the gel was cut out that corresponded to RNA of 16–30 nucleotides, as judged using a standard oligonucleotide marker. After size fractionation, of the sRNA, a ligation step was carried out. A single-stranded DNA 5′ adaptor, followed by a 3′ adaptor, was ligated to the small RNAs. The ligation products (70–90 nucleotides) were purified using urea-PAGE. The adaptors act as primer binding sites for reverse transcription and PCR amplification. Adaptor-ligated sRNAs were reverse-transcribed and amplified by PCR. The resulting cDNA tag libraries were sequenced with an Illumina genome analyzer.

### Analysis of deep sequencing data

The total sequences from the normoxic and hypoxic samples numbered 12,577,383 and 7,110,799, respectively. After 3′ adaptor removal and length range filtering (16–35), 7,297,894 and 5,256,222 sequences, respectively, remained. The following databases were used in the analysis of our deep sequencing data:

A. Mature miRNAs: miRBase, release 20.
B. ncRNAs: Ensembl "Homo_sapiens.CRCh37.69.ncrna.fa.red" (includes precursor miRNAs and other ncRNAs).
C. piRNA: piRNA Bank.
D. RNA database: NCBI FTP site (includes rRNAs and mRNAs).
E. Exons and intergenic/intronic sequences: obtained through in-house built perl script using the reference contig files from the NCBI FTP site. A detailed analysis of the deep sequencing data is given in Additional file 1.

### Expression pattern of known MiRNAs

To obtain the expression pattern of known miRNA, sequences from both samples were matched against known mature miRNA databases using an in-house built shell script. No mismatch was allowed, and sequences showing 100% similarity to a database entry were considered candidates for known mature miRNAs (Additional file 2).

### Data normalization

Normalization is required to make data comparable across experiments and to reduce the impact of non-biological variability. We performed "transcript parts per million" (TPM) and "reads per kilobase per million" (RPKM) normalization for our samples. To calculate TPM, the number of reads or frequency of the sequence are divided by total clone count of the sample and multiplied by 10^6^ while to calculate RPKM, TPM values are divided by the nucleotide length of the mature miRNA. Total clone count is the sum of the frequencies of all sequences present in the sample.

### Differentially expressed MicroRNAs

Known miRNAs expressed in normoxic sample were compared to those from hypoxic sample. A fold change of 1.5 was set as the minimum threshold to count as differential expression. A list of differentially expressed miRNAs is given in Additional file 3.

### Identification of novel MiRNAs

Sequences from both samples were matched against the above mentioned already existing and compiled databases, from mature miRNAs to intronic sequences. An elimination pipeline was used in the prediction process for novel miRNAs [27]. An elimination pipeline perl script was used for the alignments, and a mismatch of up to 2 nucleotides was allowed. Sequences that exactly matched intergenic/intronic regions were extracted along with 70 nucleotides flanking on either side. These sequences served as potential precursor miRNAs. The sequences were then analysed with the miRNA prediction algorithm tool CID-miRNA [28]. Folded precursors predicted by this program were then checked for the presence of sRNA. After this, those hairpins were considered as potential precursor, in which the mature sequence arises from the stem portion and not from the loop part. This prediction was further checked with MiPred [29]. The list of novel miRNAs is given in Additional file 4.

### IsomiRs of novel MiRNAs

A list of all the predicted novel miRNA precursors was created. Sequences that differed from the representative mature miRNA by a few nucleotides at the 5′- or 3′-end were listed as isomiRs. A representative mature miRNA was selected on the basis of presence of highest number of sequence reads. IsomiRs of miR-210 were identified in an equivalent way.

### Microarray expression profiling and analysis

U87MG cells were kept under normoxia (21% O_2_) or hypoxia (0.2% O_2_) for 48 h. Two technical replicates of total RNA were harvested and sent for microarray expression profiling (Genotypic Technology, Bangalore). The miRNA expression data were generated using Agilent Human miRNA 8 × 15 k arrays. The normalization was done using the software GeneSpring GX 11.5. Fold differences are provided in terms of log base 2. A ± 0.6 fold change (log base 2) cut-off was used for identifying differentially expressed miRNAs (Additional file 5).

### MiRNA and mRNA quantitation

Candidate miRNAs were reverse-transcribed into cDNA using specific stem-loop RT primers (Additional file 6). Quantitation was done with a CFX96™ real time PCR system (Bio-RAD) using a cDNA-specific forward primer and a universal reverse primer, as listed in Additional file 6. RNU6B was used for normalization for all samples. A list of primers along with their sequences is given in Additional file 6.

To determine the transcript levels of HIF1A, HIF3A, VEGF and CA9, total RNA was reverse-transcribed into cDNA with the RevertAid first strand cDNA synthesis kit (Fermentas), using an oligo-dT primer. The cDNA was further amplified with gene specific primers using the Fermentas SYBR Green PCR master mix. GAPDH was used for normalization.

### Target prediction of MiR-210-3p and differentially regulated novel MiRs

Potential targets of miR-210-3p were predicted using established target prediction programs. Targets were only further considered when predicted by at least three out of 11 target prediction programs (PITA, PicTar, miRanda, mirTarget2, TargetScan, NBmirTar, RNAhybrid, MicroInspector, MiTarget, RNA22 and DIANA MicroT), using the online software miRECORDS [30]. Potential targets of differentially regulated novel miRs were predicted by the TargetScan Human 5.2 custom program. A list of targets, along with their description, is given in Additional file 7.

### Prediction of HREs in the promoter of MiRNAs

The miRNAs found to be up-regulated by hypoxia (based on deep sequencing or microarray profiling) were searched for the presence of HREs within the promoter region (within 5 kb upstream of the 5′ends of pre miRNAs). The upstream region of these miRNAs was extracted from the Ensembl genome browser. HREs were predicted with program PROMO [31, 32]. PROMO uses version 8.3 of the TRANSFAC database. A list of predicted HREs is given in Additional file 8.

### Construction of 3ʹ-UTR-luciferase or promoter-luciferase constructs

To determine whether miR-210-3p down-regulates target transcripts through direct binding to the 3ʹ untranslated region (UTR), the fragment of 3ʹ UTR of the target gene [hypoxia inducible factor 3A (HIF3A)] containing the miR-210-3p binding site was PCR-amplified and cloned into a luciferase reporter vector (pMIR-Report) downstream of a firefly luciferase gene. To further test whether the predicted target HIF3A is the direct target of miR-210-3p, the miR-210-3p binding site in the 3ʹ UTR of the HIF3A gene was mutated by site-directed mutagenesis, and luciferase activity was determined. All clones were confirmed by PCR, restriction digests and sequence analysis.

### Dual luciferase assay

For luciferase assays, U87MG cells (5 × 10^4^ cells/well) were co-transfected with the 3ʹ UTR luciferase constructs along with vectors pBABE or pBABE-miR-210, using Lipofectamine 2000. pRL-TK was co-transfected in all wells for normalization of transfection efficiency. The activities of firefly (*Photinus pyralis*) and renilla (*Renilla reniformis*) luciferases were quantified 48 h post-transfection, using a dual luciferase reporter assay kit (Promega).

## Results

### MicroRNA signature of hypoxia in GBM

Though there are several reports on aberrant expression of miRNAs in hypoxic tumors, as determined by Next Generation Sequencing or microarrays, but none in GBM cells so far. Here, we present for the first time the small RNA transcriptome of U87MG, a model GBM cell line for the study glioma, and its aberrant miRNA expression profile under hypoxic conditions.

The U87MG cell line was exposed to normoxia (21% O_2_) or hypoxia (0.2% O_2_) for 48 h. RNA was then harvested, processed (see above) and analysed by deep sequencing using the Illumina platform. A detailed analysis of the sRNA deep sequencing data with respect to read lengths, annotations, expression patterns and a list of highly expressed miRNAs and piRNAs are given in Additional files 1 and 2. The expression patterns of miRNA clusters were also investigated and, in agreement with the recent literature, miRNA genes present within the same cluster showed huge variability in their expression levels in GBM cells (Additional file 1).

**Figure 1:**
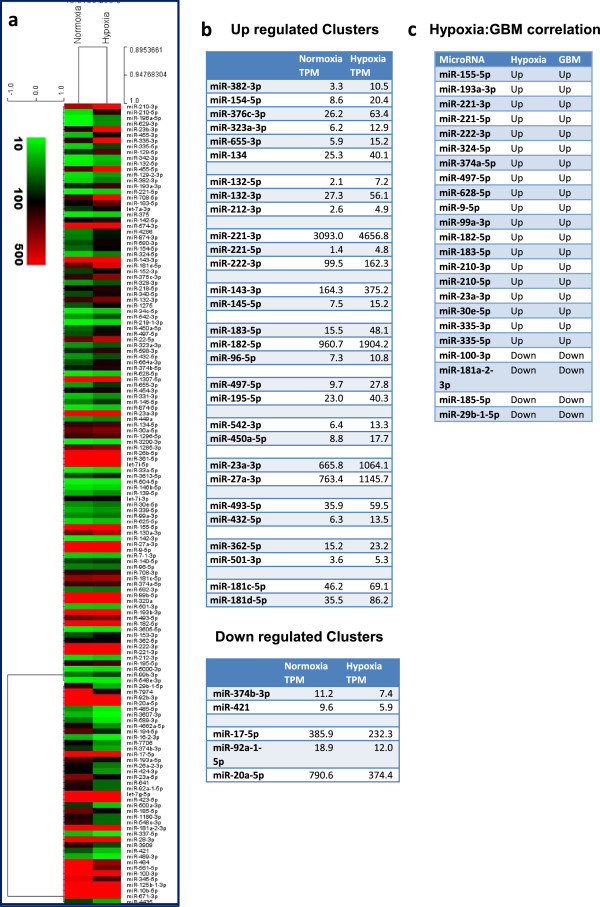
**Hypoxia regulated miRNAs.** Hierarchical clustering of hypoxia-induced and down-regulated miRNAs (>1.5-fold) in response to hypoxia (0.2% O_2_) in cell line U87MG **(a)**. List of hypoxia-regulated miRNA clusters in U87MG cells **(b)**. A table showing correlation of microRNAs altered in hypoxia or in GBM tumor tissues **(c)**.

The deep sequencing data were normalized according to TPM or RPKM, as described in the "Methods". A comparison of the normalized normoxic and hypoxic profiles of U87MG cells identified many differentially expressed miRNAs (>1.5 fold regulated). A total of 141 miRNAs were found to be differentially regulated (102 up-regulated and 39 down-regulated) in response to hypoxia (Figure 1a and Additional file 3). The highly up-regulated miRNAs include miR-210-3p/5p, miR-196a-5p, miR-629-3p, miR-23b-3p, miR-455-3p/5p, miR-335-3p/5p, miR-129-5p/3p, miR-342-3p, miR-132-5p, miR-382-3p, miR-193a-3p, miR-221-5p, miR-708-5p and miR-183-5p (Figure 1a and Additional file 3). These miRNAs showed a more than 3-fold higher expression in hypoxic cells and are among the top twenty up-regulated miRNAs. Notably, miR-210, a miRNA shown to be hypoxia up-regulated in several cell lines of various tissues, showed highest induction of both miR-210-3p (25-fold) and miR-210-5p (12-fold) in response to hypoxia.

Among the down-regulated miRNAs, miR-29b-1-5p, miR-7974, miR-3607-3p, miR-589-3p, miR-92b-3p, miR-485-5p, miR-4662a-5p, miR-16-2-3p, miR-20a-5p and miR-194-5p were found to be down-regulated by more than 2-fold in hypoxic cells (Figure 1a and Additional file 3).

We next checked which members of any miRNA cluster were co-regulated. Interestingly, we found that 29 miRNAs were upregulated as part of 11 miRNA clusters while members of the miR-374b cluster and miR-17/92 cluster were down-regulated (Figure 1b).

We compared the published GBM tumor and normal brain miRNA signatures with the GBM hypoxia miRNA signature and interestingly found that several of the hypoxia-induced miRNAs were also overexpressed in GBM, suggesting that the GBM-associated miRNA profile may have a hypoxia signature [33, 34] (Figure 1c).

We also conducted miRNA microarray profiling of U87MG cells grown in normoxia (21% O_2_) or hypoxia (0.2% O_2_) for 48 h. A total of 10 microRNAs were found to be up regulated and 23 microRNAs were found to be down regulated in hypoxia [>0.6-fold (log base 2), p < 0.05] (Additional file 5). A comparison of both microarray and deep sequencing data found miR-210-3p and miR-1275 to be up-regulated and miR-10b-5p, miR-181a-2-3p and miR-185-5p to be down-regulated, according to both data sets.

**Figure 2:**
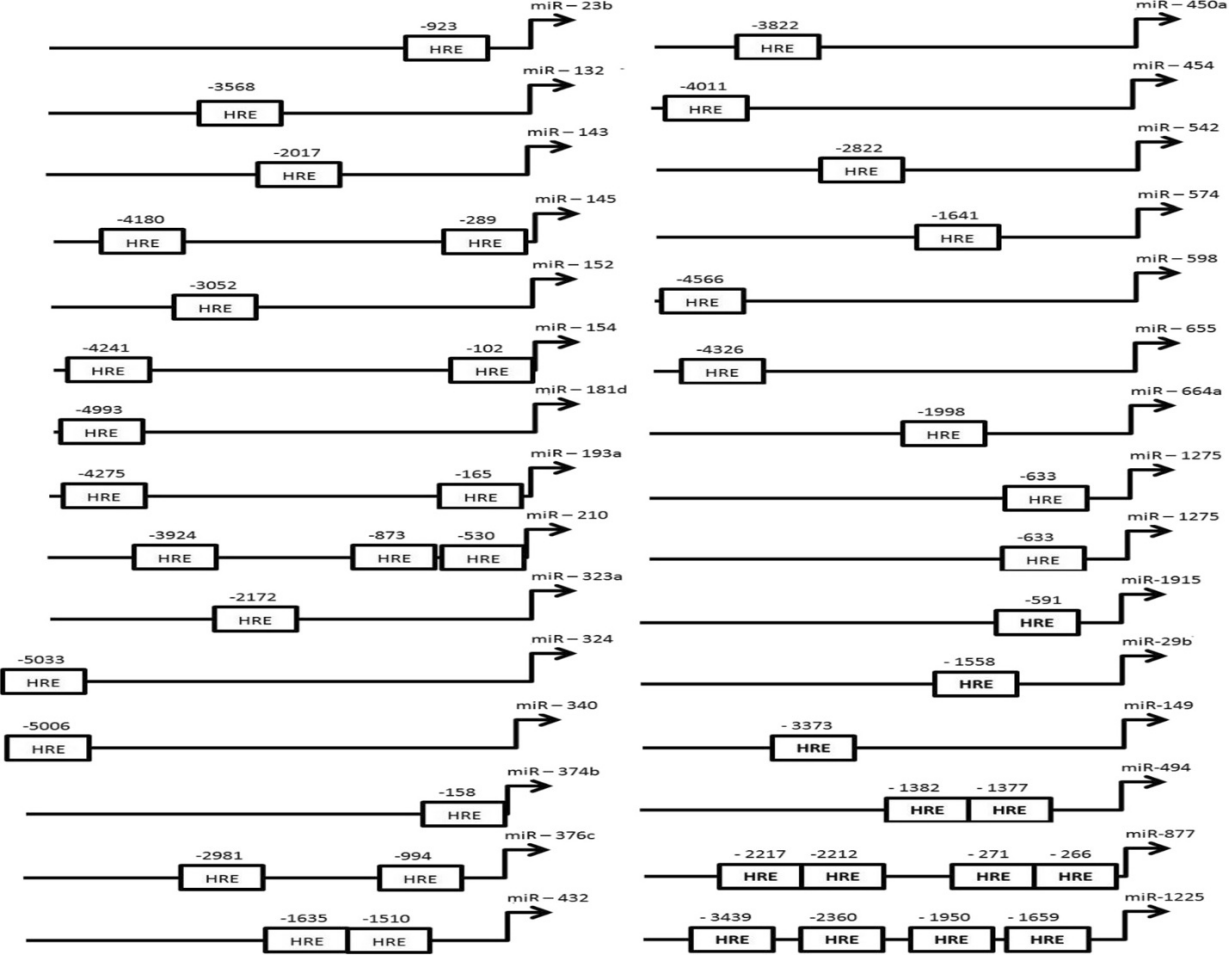
**Figure showing location of HREs in the promoters of hypoxia-induced miRNAs, predicted by the program PROMO.**

Generally, hypoxia-regulated genes or miRNAs bear HREs in their promoters, to which a transcription factor called HIF1 binds, which induces their expression [19]. We therefore looked for the presence of HREs within the promoter regions of those miRNAs upregulated by hypoxia. A total of 5 kb upstream of these miRNAs was extracted, and HREs were predicted using the prediction program PROMO (details in the Methods). A total of 30 upregulated miRNA genes were found to contain one or more HREs (Figure 2 and Additional file 8).

**Figure 3:**
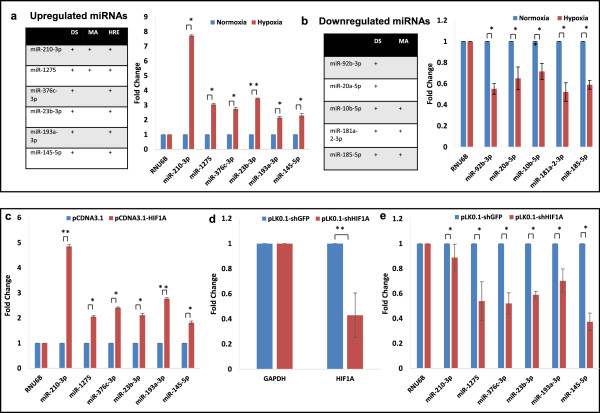
**Quantitative RT-PCR data showing miRNA levels in response to hypoxia or HIF1A.** Graph showing miRNAs that are upregulated **(a)** or downregulated **(b)** in response to hypoxia. **(c)** U87MG cells were transfected with pCDNA3.1or a HIF1A over-expressing plasmid (pCDNA3.1-HIF1A), and miRNA levels were determined. U87MG cells were transfected with pLK0.1-shGFP or a shHIF1A over-expressing plasmid (pLK0.1-shHIF1A) and levels of HIF1A **(d)** and miRNAs **(e)** in response to hypoxia were determined. The graphical data points represent mean ± S.D. of at least three independent experiments. (*P > 0.01 and < 0.05; **P < 0.01). Error bars denote ± S. D.

We next validated the expression of the differentially regulated candidate miRNAs identified by deep sequencing or microarray profiling or both, using quantitative stem loop RT-PCR, which is used for detection of mature miRNAs (Figure 3a, b). Among the upregulated miRNAs, an additional criterion of presence of HREs in their promoter was considered for their validation. Notably, miR-210-3p was found to be most induced on the basis of qRT-PCR data too. To check for the universality of differential expression of miRNAs in normoxic v/s hypoxic cells, the results were further verified for cell line U251MG. Out of 11 miRNAs tested, eight showed a similar expression pattern as in U87MG cells, with miR-210-3p showing the highest induction (Additional file 9). However, expression of miR-376c-3p, miR-193a-3p and miR-10b-5p did not change significantly in response to hypoxia in U251MG cells, as opposed to U87MG. These differences could be due to several factors, including genotypic differences in the two cell lines, which are derived from two different GBM patients.

Often miRNAs exist in alternative forms, called "isomiRs" that differ from each other in terminal nucleotides. We analysed if the isomiR profile of miR-210 changes in normoxic versus hypoxic samples. A total of 320 isomiR species were detected, of which the normoxic samples expressed only 73, while the hypoxic samples expressed 312 isomiRs, of which 175 were singletons (Additional file 10). While the reference miR-210 sequence from miRBase was most abundant (Nor-396 and Hyp-7252 reads), several other miR-210 isomiRs also showed high expression levels, ranging from 113 to 699 among the highly expressed hypoxia sequences.

### Hypoxia-inducible factors (HIFs) and their role in miRNA induction

HIFs are transcription factors that respond to hypoxia in the cellular environment [35]. Promoter regions of HRM genes contain HREs that are bound by HIF1 in response to hypoxia [19]. Many HRMs, such as miR-210 [19], -155 [36], -373 [37] have been shown before to bear HREs in their promoter regions and are dynamically regulated by HIF1 in response to hypoxia.

To evaluate HIF1-mediated induction of hypoxia-regulated miRNAs, U87MG cells were transfected with either a control (pCDNA3.1) or HIF1A-overexpressing plasmid (pCDNA3.1-HIF1A). Since HIF1A is degraded under normoxia, we expressed a HIF1A mutant (proline to alanine mutations) that is extremely stable. Interestingly, miRNAs upregulated under hypoxia and containing HREs showed a higher expression in HIF1A transfected cells than in control cells. MiR-210-3p showed an about 5-fold and miR-1275, miR-376c-3p, miR-23b-3p, miR-193a-3p and miR-145-5p showed an about 2-fold induction in HIF1A transfected cells (Figure 3c). In parallel, we transfected U87MG cells with a control plasmid (pLK0.1-shGFP) or a shHIF1A over-expressing plasmid (pLK0.1-shHIF1A) and exposed the cells to hypoxia (0.2% O_2_) for 48 h. The transcript level of HIF1A was determined and was found to be down-regulated up to 2.4-fold compared to control cells in hypoxia (Figure 3d). As expected, down-regulation of HIF1A by using specific shRNA inhibited hypoxia-mediated induction of these miRNAs (Figure 3e). Overall, these results show that expression of miR-210-3p, miR-1275, miR-376c-3p, miR-23b-3p, miR-193a-3p and miR-145-5p is HIF-1 dependent.

### MiR-210-3p promotes HIF transcriptional activity

**Figure 4:**
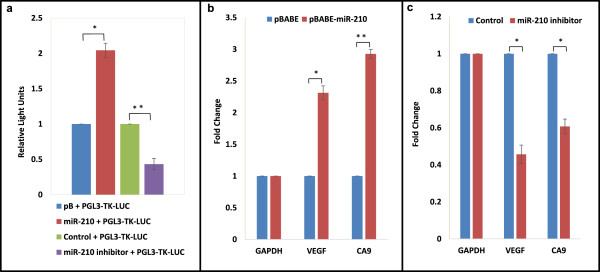
**MiR-210-3p induces HIF transcriptional activity.** U87MG cells were transfected with a HRE luciferase vector, along with either miR-210-3p overexpression - [(pBABE-miR210) or control (pBABE)] or inhibition - [(miR-210 inhibitor) or (control)] vectors, and HRE transcriptional activity was assayed **(a)**. U87MG cells were transiently transfected with either a miR-210-3p over-expression vector (pBABE-miR-210) or the empty pBABE-puro parent vector **(b)** or with either a miR-210-3p inhibitor or control oligos **(c)**, and VEGF/CA9 levels were determined by qRT-PCR. The graphical data points represent mean ± S.D. of at least three independent experiments. (*P > 0.01 and < 0.05; **P < 0.01). Error bars denote ± S. D.

We also tested if a feedback loop of the miR-210-3p and HIF pathways exists. MiR-210-3p levels were modulated under hypoxia, and HIF transcriptional activity was measured using a control HRE luciferase vector. Interestingly, miR-210-3p overexpression led to an increase in HRE activity, while miR-210-3p inhibition led to a decrease (Figure 4a). This shows that miR-210-3p promotes HIF transcriptional activity, which was also reflected in the increase in expression of the HIF target genes VEGF and CA9 in response to miR-210-3p over-expression and in their down-regulation in response to miR-210-3p inhibition (Figure 4b, c).

### MiR-210-3p a putative novel hypoxia marker in GBM patients

Hypoxia is a critical aspect of the glioma microenvironment, and it has been associated with poor prognosis [38]. Thus, the hypoxic state of GBM tumors is often measured to predict patient treatment response [39]. Also, identifying hypoxia-related molecular targets may be useful to develop novel treatment approaches.

**Figure 5:**
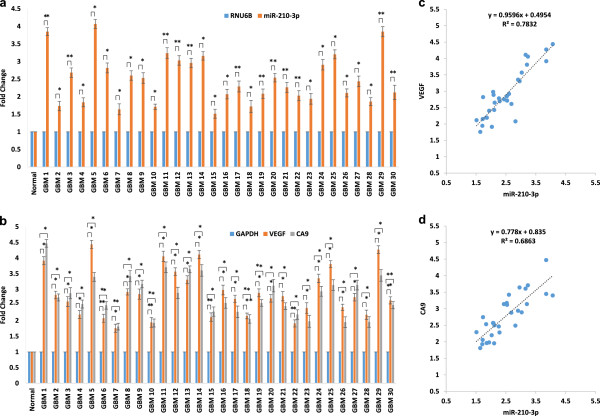
**Quantitation and correlation of miR-210-3p levels with hypoxia markers VEGF and CA9.** The graph showing levels of miR-210 **(a)** and VEGF, CA9 **(b)** in normal and GBM tumor tissue (n = 30) samples using qRT-PCR. Correlation between miR-210-3p and VEGF **(c)** and miR-210-3p and CA9 **(d)** in GBM tumor tissues. The miR-210-3p levels have been normalized with RNU6B, while GAPDH was used for VEGF and CA9 normalization. The graphical data points in a and b represent mean ± S.D. of at least three independent experiments. (*P > 0.01 and < 0.05; **P < 0.01). Error bars denote ± S. D.

Since miR-210-3p is highly up-regulated in hypoxic GBM cells, as identified by deep sequencing and other detection methods like microarray and quantitative stem loop RT-PCR, its level was assessed in 30 GBM patient samples, and efforts were made to correlate the levels with known hypoxia marker genes in GBM. Notably, miR-210-3p was highly expressed (>1.5 fold, p < 0.05 in 30/30; > 2.0 fold, p < 0.05 in 22/30) GBM patient samples as compared to normal control (Figure 5a). Further, transcript levels of hypoxic markers VEGF and CA9 were also checked in patients, and normal samples and correlations were tested between miR-210-3p and VEGF/CA9 levels. We found that 27 out of 30 GBM samples showed a > 2 fold increased VEGF/CA9 expression level compared to normal, suggesting their high hypoxic content (Figure 5b). Interestingly, miR-210-3p levels were found to be highly correlated (Pearson correlation coefficient > 0.8), with both hypoxic markers, suggesting that miR-210-3p is regulated by hypoxia in GBM tumor tissues and may serve as an intrinsic hypoxia marker (Figure 5c, d).

### MiR-210-3p promotes hypoxia/stress/chemo-resistance in GBM

**Figure 6:**
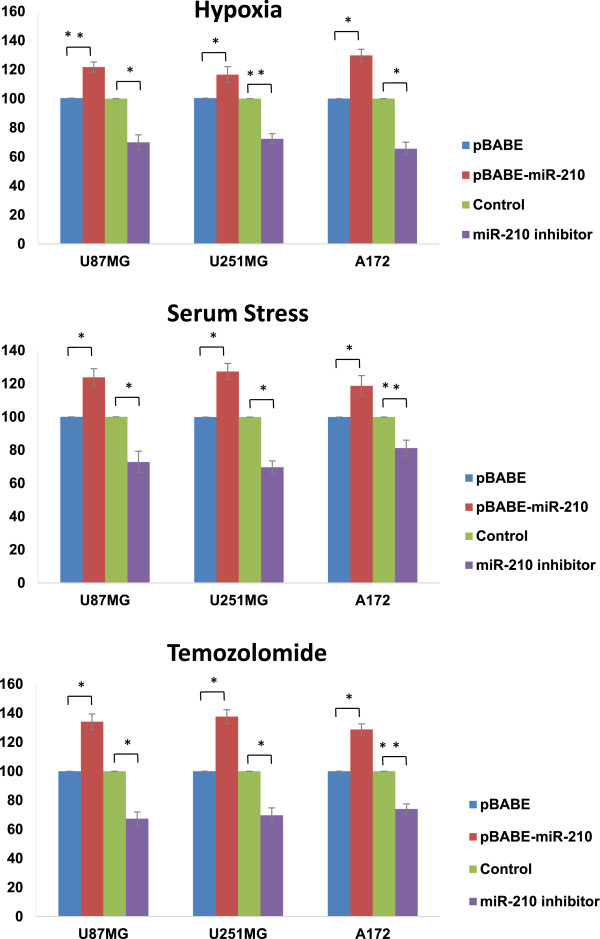
**MiR-210-3p functions.** Graphs showing MTT assay results of cell survival on the 3^rd^ day in U87MG, U251MG and A172 cells in response to either miR-210 overexpression - [miR-210 polyclonals (pBABE-miR-210) or control (pBABE)] or inhibition - [(miR-210 inhibitor) or (control)] under **(a)** 0.2% hypoxia **(b)** or serum starvation or **(c)** chemo drug temozolomide treatment. The graphical data points represent mean ± S.D. of at least three independent experiments. (*P > 0.01 and < 0.05; **P < 0.01). Error bars denote ± S. D.

Several hypoxia-regulated microRNAs have been reported to modulate survival, growth advantage and therapy resistance of cancer cells [40]. We thus investigated whether miR-210-3p plays a similar role. MiR-210-3p over-expression and silencing strategies were used to analyse its functions. Quantitative RT-PCR data show that miR-210-3p was ~ 4.2 fold up-regulated in miR-210-3p over-expressing U87MG cells (Additional file 11a), while miR-210-3p was down regulated up to ~5.6 fold in U87MG cells transiently transfected with a miR-210-3p inhibitor (Additional file 11b). Interestingly, miR-210-3p overexpressing U87MG, U251MG and A172 cells showed better survival in tumor microenvironment conditions, i.e. hypoxia and serum starvation, while the GBM cells transfected with the miR-210-3p inhibitor showed the opposite trend (Figure 6a, b). Moreover, miR-210-3p over-expressing GBM cells showed increased resistance to temozolomide (a routinely used drug for GBM patients) mediated death while miR-210-3p inhibition made cells more sensitive (Figure 6c). Overall, miR-210-3p seems to promote aggressiveness and therapy resistance of GBM cells.

### MiR-210-3p targetome

**Figure 7:**
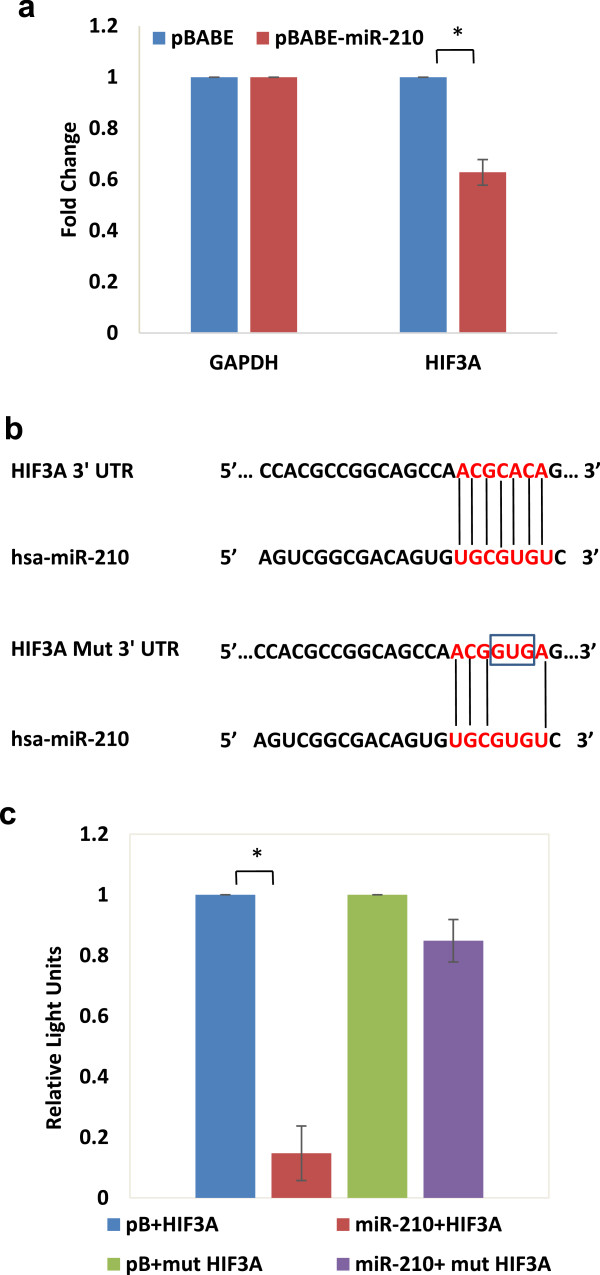
**MiR-210-3p targets HIF3A.** Effect of transient over-expression of miR-210-3p on HIF3A transcript levels was determined by qRT-PCR **(a)**. Diagram showing wild type/mutated miR-210-3p binding site in the HIF3A 3′ UTR **(b)**. 3′ UTR luciferase results obtained by cotransfection of luciferase constructs bearing wild type/mutated miR-210-3p binding sites in the HIF3A 3′ UTR, along with miR-210-3p over-expression vector (pBABE-miR-210) or the empty pBABE-puro parent vector **(c)**. The graphical data points in a and c represent mean ± S.D. of at least three independent experiments. (*P > 0.01 and < 0.05; **P < 0.01). Error bars denote ± S. D.

MicroRNAs exert their effect either by binding directly to the 3′ UTRs of target transcripts, or indirectly by inhibiting the expression of transcription factors or regulators. We performed *in silico* searches, as described in the "Methods", to identify target transcripts of miR-210-3p (Additional file 7). Interestingly, HIF3A, a negative regulator of hypoxic response was identified as putative target of miR-210-3p by three target prediction programs (PITA, miRanda and RNAhybrid). To determine the effect of miR-210-3p on HIF3A transcript, we transiently over-expressed miR-210-3p in U87MG cells and determined the HIF3A transcript level 48 h post-transfection by qRT-PCR. We found ~ 0.6 fold downregulation at the transcript level (Figure 7a).

To further validate the results from qRT-PCR, a HIF3A 3′UTR-luciferase reporter assays were performed. Interestingly, results showed robust downregulation (~0.2 fold) in luciferase activity, suggesting that miR-210-3p binding to the HIF-3A 3′UTR leads to its down-regulation. To verify this, the miR-210-3p binding site in the HIF3A 3′UTR was mutated through site-directed mutagenesis (Figure 7b). As anticipated, only minimal inhibition was observed when the mutated 3′UTR was used, suggesting HIF3A to be a direct target of miR-210-3p (Figure 7c).

### Discovery of novel miRNAs

Deep sequencing profiling of sRNAs is a powerful tool for the identification of novel miRNAs, since this method is independent of the prior knowledge of candidate sequences. Since miRNAs mainly arise from intergenic and intronic regions of genomes, sequence reads that matched these regions were extracted along with 70 nucleotide flanking regions from both ends. Novel miRNAs were predicted using various miRNA identification algorithms, as described in the "Methods".

**Figure 8:**
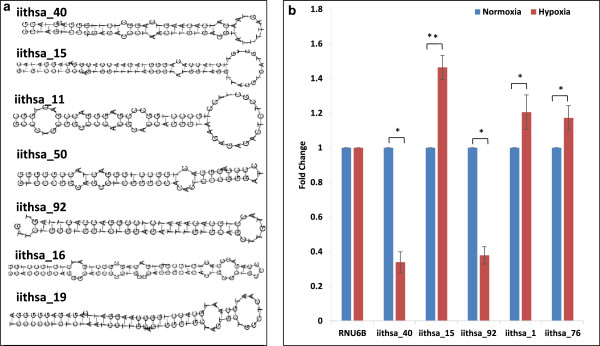
**Identification of novel miRNAs. (a)** Secondary structure prediction of highly expressed novel miRNAs using RNA-fold. **(b)** Detection of novel miRNAs through Real-time PCR in normoxic and hypoxic samples. The graphical data points in b represent mean ± S.D. of at least three independent experiments. (*P > 0.01 and < 0.05; **P < 0.01). Error bars denote ± S. D.

A total of 139 novel miRNAs were predicted, with only 14 common in both the samples. A total of 7 miRNAs also exhibited isomiRs that differed from the standard mature sequence by a few nucleotides, mostly at the 3′end (Additional file 4). A total of 7 miRNAs showed more than 10 reads. Secondary structures of these are shown, as predicted by RNAfold [41] (Figure 8a). Three of these novel miRNAs with higher read numbers were validated by quantitative stem loop RT-PCR and, in agreement with the deep sequencing reads, displayed differential expression (Figure 8b). The novel miRNAs, iithsa_40 and iithsa_92 showed an about 3-fold reduced level, while iithsa_15 showed induction of expression in hypoxic cells, as determined by real time PCR. We also performed target site analyses of these three hypoxia-regulated novel miRNAs. The target lists were compared with the hypoxia-regulated mRNA list in GBM [42] (Additional file 7). Interestingly, we found that iithsa_40 showed inverse correlation with SERPINE1, while iithsa_15 showed inverse correlation with hypoxia-regulated genes HNRNPA1 and EIF2C1. What significance these correlations have, needs to be investigated. We could further validate by qRT-PCR the expression of 2 novel miRNAs having 1–3 reads, using a higher amount of template cDNA (Figure 8b). This was particularly important to distinguish the fact whether novel miRNAs displaying 1 reads were really existent or they appear as a result of sequencing error.

## Discussion

This study sheds light on the sRNA composition of GBM cell line U87MG and its alteration in response to severe (0.2% O_2_) and chronic (48 h) hypoxia, using deep sequencing of sRNA populations. The analysis of the deep sequencing reads reveals that miRNAs form the most abundant class of sRNA (75–80%) in both normoxic and hypoxic samples. A total of 643 and 627 mature miRNAs were found to be expressed in normoxic and hypoxic cells, respectively, with a range of expression from 1 to > 10,000 reads. Interestingly, miRNAs expressed within a cluster also showed a large variation in expression. This was also reported in recent papers, based on analysis of deep sequencing reads. The reason for this variation remains unclear. Possible explanations may be the use of different promoters, different post-transcriptional processing or differences in stability of mature miRNAs. We identified several highly expressed miRNAs (>0.1 million counts) in GBM cells. A comparison with published lists of highly expressed miRNAs from other cancer cell lines shows that the highly expressed miRNA pool varies between cell lines. Secondly, some of the highly expressed miRNAs in GBM cells are known oncomiRs (miR-21, let-7a and miR-92a). A total of 31 piRNAs were found to be expressed in GBM cells, with 7 of them being differentially expressed under hypoxia. It would be interesting to see whether piRNAs play any role in the hypoxic response.

Differentially expressed miRNAs identified using deep sequencing and/or microarray hybridization were further verified by stem-loop qRT-PCR on two GBM cell lines. Subsets of these miRNAs were found to bear HREs in their promoters, similar to hypoxia-regulated protein coding gene promoters. We validated expression and HIF1A dependent induction of miR-210-3p, miR-1275, miR-376c-3p, miR-23b-3p, miR-193a-3p and miR-145-5p in U87MG using HIF1A over-expression and silencing experiments. Since recent studies highlighted specific miRNAs that may directly or indirectly control HIF1A levels and hypoxia-inducible gene expression, we cannot rule out the existence of a positive or negative feedback loop in relation to these HRMs. For example, miR-210-3p represses glycerol-3-phosphate dehydrogenase 1-like (GPD1L) which, in turn, stabilizes HIF-1A by reducing hyper-hydroxylation [43]. Interestingly, our results identify HIF3A (a negative regulator of hypoxic response) as direct target of miR-210-3p. It has been shown before that ectopic expression of HIF3A results in down-regulation of VEGF and results in reduced vascular density of tumors and slower tumor growth *in vivo* [44]. Thus, it is possible that miR-210-3p, through downregulation of HIF3A, may be part of a novel mechanism of positive feedback regulation of angiogenesis and maintenance of a vascular phenotype in GBM. In addition, we see that miR-210-3p over-expression also leads to induction of HIF transcriptional activity and then also of its targets VEGF and CA9. In contrast, miR-210-3p-inhibition under hypoxia prevents HIF mediated induction of VEGF and CA9. Overall, these results suggest that miR-210-3p is involved in induction of the HIF pathway.

In contrast, some miRs, such as the miR-17-92 cluster [45] and miR-519 [46] have been shown to down-regulate the HIF1A transcript. The possibility of regulation of HRMs by other transcription factors like TWIST, peroxisome proliferator-activated receptors γ (PPARγ), or GATA1, also exists [47–49]. Several HRMs like miR-210, -23b, -335 and -193a have been reported to be hypoxia-inducible in other cell lines too [19, 50, 23]. However, whether hypoxia-mediated regulation of miRNAs like miR-1275,-708,-129,-455 is GBM-specific or occurs in other cell types too needs to be investigated.

Overall, miR-210 showed the highest induction (~25-fold - deep sequencing, ~ 3-fold - microarray, ~8-fold - qRT-PCR) in response to hypoxia. Interestingly, we also noticed a > 4 fold induction of miR-210 isomiR species in the hypoxic sample (312), compared to the normoxic one (73). Several of these isomiRs also showed differential expression under hypoxia. The isomiR generation has been correlated to differential processing by miRNA processing enzymes Drosha and Dicer [51]. Whether, dicer processivity is altered under hypoxia in GBM remains to be seen. Another possibility is that certain nucleotide transferases become activated under hypoxia and add nucleotides to certain miRNAs. What effect these additions have on miRNA stability or target binding needs further investigation.

Though miR-210-3p has been well established as a HRM/hypoxamiR in several cancers, its role in the context of GBM remains unclear. Our findings indicate that miR-210-3p functions as an oncomiR in GBM. We show that high miR-210-3p-levels in GBM promote cell survival in the tumor microenvironment (under hypoxia and nutrient deprivation) and provide the cells with resistance to temozolomide mediated death. Whether survival as measured by the MTT assay involves miR-210-3p-mediated increased cell proliferation or decreased apoptosis remains to be seen. In other studies, miR-210 has been shown to modulate mitochondrial oxygen uptake and create a pseudo-hypoxic environment [52], or to act as an oncomiR by repressing the Myc antagonist Max-binding protein [53] (MNT) and as pro-angiomir by targeting ephrin-A3 [54] in other cancers. A recent publication also shows miR-210-3p as a hypoxia-regulated miRNA in a GBM cell line [55]; however, its function remains unexplored. Overall, which role these HRMs play in GBM pathogenesis has not been elucidated. Based on the current literature, some of them act as oncomiRs and promote drug resistance in GBM. MiR-23b is highly expressed in GBM tumors and its up regulation is correlated with tumor survival [56]. Similarly, overexpression of miR-335 is associated with poor prognosis in human glioma [57]. MiR-455 promotes acquired temozolomide resistance in GBM [58]. MiR-193a is associated with poor survival in GBM [59]. MiR-183 up-regulates HIF-1A by targeting isocitrate dehydrogenase 2 [60]. On the other hand, hypoxia-down-regulated miR-29b inhibits invasion and proliferation of GBM [61]. Overall, these observations are in line with the current understanding that hypoxia promotes GBM aggressiveness and these HRMs may play a prominent role in it.

We compared our hypoxia-regulated miRNA list with published lists of miRNAs that are known to be deregulated in GBM tumor tissues. Interestingly, we found that hypoxia-induced microRNAs such as miR-221, 222, miR-155, miR-210, etc., are also overexpressed in GBM tumor tissues, suggesting that hypoxia may be one of the major factors contributing to their elevated expression.

A particularly interesting finding of our study is a positive linear correlation of the level of miR-210-3p with that of hypoxic markers (VEGF, CA9) in GBM patient samples. This suggests that the level of miR-210-3p in GBM tumors depends on the *in vivo* O_2_ concentration in the tumor. Considering the more stable nature of miRNAs compared to the more labile mRNAs, miR-210-3p may show a better potential as intrinsic hypoxia marker for GBM tumor samples than VEGF and CA9 (7,8). Also, target gene analyses of miR-210-3p in these tumor samples may hint at pathways responsible for hypoxia-resistance in GBM.

We discovered a total of 139 novel miRNAs in U87MG cells, using predictions of programs CID-miRNA and miPred. We validated the expression of five novel miRNAs and found three to be differentially expressed. The functional role of these novel miRNAs is not yet known. Importantly, the detection of two novel miRNAs, showing only 1–3 reads in either sample proves that these single reads are not mere sequencing errors, but rather very low abundant novel miRNAs. Specific novel miRNAs, also showed the presence of isomiRs. The isomiRs carried mostly extra nucleotides at their 3′ends, suggesting generation as a result of inappropriate DICER processing.

## Conclusions

This study is a first effort to identify hypoxic signatures of miRNA in GBM, using deep sequencing and microarray technologies. We identify miR-210-3p as a HIF regulated miRNA that acts as critical mediator of the hypoxic response and thus as an oncomiR in GBM. The regulatory and functional characterization of other identified known and novel HRMs may help with the identification of novel hypoxia biomarkers and therapeutic targets for GBM.

### Consent

Written informed consent was obtained from the patient for the publication of this report and any accompanying images.

## Electronic supplementary material

Additional file 1: **A detailed analyses of the sRNA deep sequencing data.** Read lengths, annotations, expression patterns, miRNA cluster analyses and a list of highly expressed miRNAs and piRNAs are given. (DOCX 92 KB)

Additional file 2: **The known miRNA expression pattern in response to normoxia (Sheet 1) and hypoxia (Sheet 2).** (XLSX 46 KB)

Additional file 3: **List of up-regulated (Sheet 1) and down-regulated (Sheet 2) miRNAs showing > 1.5-fold difference in response to hypoxia in U87MG cells, as determined by deep sequencing.** The data were normalised using TPM or RPKM for each sample. (XLSX 25 KB)

Additional file 4: **List of the predicted novel miRNAs with their isomiR sequences.** (XLSX 24 KB)

Additional file 5: **List of up-regulated (Sheet 1) and down-regulated (Sheet 2) miRNAs showing > 0.6 fold (log base 2) difference in response to hypoxia in U87MG cells, as determined by microarray profiling.** (XLSX 359 KB)

Additional file 6: **List of primers used for detection of specific miRNAs and mRNAs with Quantitative RT-PCR and for cloning wild type/mutated 3′ UTR of HIF3A.** (DOCX 18 KB)

Additional file 7: **List of the predicted targets of miR-210-3p and differentially regulated novel miRs.** The prediction was done using the program miRECORDS. (XLSX 144 KB)

Additional file 8: **List of HREs predicted in the promoter of miRNAs up-regulated in response to hypoxia, determined by deep sequencing and microarray profiling.** The prediction was done using the program PROMO. (DOCX 15 KB)

Additional file 9: **Quantitative RT-PCR data showing miRNA levels in response to hypoxia.** Graph showing miRNAs upregulated (a) or downregulated (b) in response to hypoxia in U251MG cells. The graphical data points represent mean ± S.D. of at least three independent experiments. (*P > 0.01 and < 0.05; **P < 0.01). Error bars denote ± S. D. (PPTX 73 KB)

Additional file 10: **List of predicted isomiRs of miR-210-3p in normoxic and hypoxic U87MG cells, as determined by deep sequencing.** (XLSX 19 KB)

Additional file 11: **Quantitative RT-PCR data showing the miR-210-3p level in a miR-210-3p overexpressing U87MG cell line or control U87MG cells (a) and U87MG cells transiently transfected with miR-210-3p inhibitor or control oligos (b), respectively.** The graphical data points represent mean ± S.D. of at least three independent experiments. (*P > 0.01 and < 0.05; **P < 0.01). Error bars denote ± S. D. (PPTX 60 KB)

## Abbreviations

HIF1A: Hypoxia inducible factor 1 A
CA9: Carbonic anhydrase 9
VEGF: Vascular endothelial growth factor
piRNA: Piwi interacting RNA
TPM: Transcripts parts per million
RPKM: reads per kilobase per million
GAPDH: Glyceraldehyde 3-phosphate dehydrogenase
lincRNA: Long intergenic non-coding RNA
snRNA: Small nuclear RNA
snoRNA: Small nucleolar RNA
scRNA: Small cytoplasmic RNA
HRM: Hypoxia regulated miRNA
HREs: HIF1A response elements.
