# Supplementary material for: Hypoxic signature of microRNAs in glioblastoma: insights from small RNA deep sequencing
Source: BMC Genomics. 2014 Aug 17;15(1):686. doi: 10.1186/1471-2164-15-686 (PMC4148931; doi:10.1186/1471-2164-15-686)
Supplement: Supplementary file 1 — Additional file 1: A detailed analyses of the sRNA deep sequencing data. Read lengths, annotations, expression patterns, miRNA cluster analyses and a list of highly expressed miRNAs and piRNAs are given. (DOCX 92 KB) [file 12864_2014_6378_MOESM1_ESM.docx]

**Small RNA transcriptome of Glioblastoma cell line**

Sequencing reads derived from the sequencing data showed a good size distribution of sRNAs with most reads falling in 21 to 24 nucleotide long range **(Figure 1)**.

**Figure 1: Size distribution graph: A size distribution graph of sRNA reads obtained after deep sequencing sRNA population of normoxic [21% O_2_] (a) and hypoxic [0.2% O_2_] (b) U87MG cells.**

A total of 5.8 and 4.4 million reads were in this range in normoxic and hypoxic cells, respectively suggesting the expression data to be enriched in miRNAs. Sequencing reads were matched against databases of known mature miRNAs (miRBase version 20) and other RNAs (tRNA, sn/snoRNA, rRNA, mRNA, piRNA, lincRNA and scRNA) **(Figure 2)**.

**Figure 2: Expression level of small RNAs. A pie chart showing frequency of different classes of RNA species present in sRNA libraries of normoxic [21% O_2_] and hypoxic [0.2% O_2_] U87MG cell line.**

As expected miRNAs form the most abundant class of sRNA (75-80%) in both the samples. A total of 643 and 627 mature miRNAs were found to be expressed in normoxic and hypoxic cells, respectively. The expression levels of miRNAs span from less than 10 to more than 10^4^ counts **(Figure 3)**.

**Figure 3. Expression of known miRNAs is represented in the form of frequency count v/s no. of miRNAs in normoxia and hypoxia samples.**

The highly expressed miRNAs (>0.1 million reads) in both normoxic and hypoxic samples include miR-21-5p, miR-22-3p, let-7a-5p and miR-92a-3p **(Figure 4a)**. On contrary, several miRNAs were expressed poorly having <10 counts **(Figure 4b)**. Further, reads that matched with the intergenic and intronic regions served as a source of novel miRNAs.

**b**

**a**

**Figure 4. The abundance of highly expressed [>10,000 counts] (a) and low expressed [<10 counts] (b) select miRNAs in normoxic and hypoxic cells.**

A total of 31 piRNAs were found to be expressed with 18 common in normoxic and hypoxic samples. While most piRNAs exhibit reads more than 1, the expression range varies from 1 to 1868. An up regulation was seen in hsa_piR_001311, hsa_piR_016926, hsa_piR_018569, hsa_piR_020541, hsa_piR_019628 and hsa_piR_001101 while hsa_piR_016659 displayed down regulation in hypoxia **(Table 1)**.

**Table1: The known piRNA expression pattern in normoxia and hypoxia.**

**MiRNA cluster analysis**

In our study a total of 465 miRNA genes organized in 153 clusters were analyzed. However, we analyzed expression pattern of only 32 clusters since rest 121 clusters were either not expressed (reads=0) or only single miRNA was detected in the cluster. In agreement with recent reports, miRNA genes present within same cluster showed huge variability in their expression level in GBM **(Table 2)**.

| **S. No.** | **miRNA** | **Normoxia TPM** | **Hypoxia TPM** |
| --- | --- | --- | --- |
| **1** | MiR-532 | 127.4342 | 161.71302 |
|  | MiR-500a | 10.825 | 5.51727 |
|  | MiR-362 | 15.2099 | 23.2106 |
|  | MiR-501 | 3.56267 | 5.32702 |
|  | MiR-660 | 37.271 | 54.0312 |
|  | MiR-502 | 1.64431 | 1.71226 |
|  | MiR-188 | 0.274052 | 0.380501 |
|  |  |  |  |
| **2** | miR-99b | 918.2105 | 1355.1591 |
|  | let-7e | 1580.4529 | 2021.98053 |
|  | miR-125a | 1450.8322 | 1177.8376 |
|  |  |  |  |
| **3** | MiR-106b | 59.88027 | 67.15852 |
|  | MiR-93 | 570.71239 | 459.8360003 |
|  | MiR-25 | 3196.67754 | 3454.76376 |
|  |  |  |  |
| **4** | miR-493 | 60.8395 | 86.3739 |
|  | miR-337 | 9.86586 | 10.46379 |
|  | miR-665 | 0.548103 | 0.951254 |
|  | miR-431 | 10.00288 | 8.75154 |
|  | miR-433 | 0.685129 | 1.52201 |
|  | miR-127 | 3084.86077 | 4380.90527 |
|  | miR-432 | 6.30319 | 13.5078 |
|  | miR-136 | 283.780155 | 299.455003 |
|  |  |  |  |
| **5** | miR-183 | 15.4839 | 48.1334 |
|  | miR-96 | 7.26237 | 10.8443 |
|  | miR-182 | 960.688 | 1904.22 |
|  |  |  |  |
| **6** | miR-200c | 3.42565 | 4.75627 |
|  | miR-141 | 0.685129 | 0.570752 |
|  |  |  |  |
| **7** | miR-212 | 3.97375 | 6.08802 |
|  | miR-132 | 29.32349 | 63.35353 |
|  |  |  |  |
| **8** | miR-497 | 9.85 | 28.15 |
|  | miR-195 | 27.4 | 46.22 |
|  |  |  |  |
| **9** | mir-181c | 46.71 | 70.58 |
|  | mir-181d | 35.48 | 86.18 |
|  |  |  |  |
| **10** | mir-99a | 190.73 | 236.097 |
|  | let-7c | 856.137 | 1179.17 |
|  |  |  |  |
| **11** | miR-301b | 10.551 | 12.176 |
|  | miR-130b | 47.27 | 52.88 |
|  |  |  |  |
| **12** | MiR-191 | 8605.91 | 11071.1 |
|  | MiR-425 | 15.47 | 13.5 |
|  |  |  |  |
| **13** | Mir-143 | 164.42 | 375.364 |
|  | Mir-145 | 7.53642 | 15.2201 |
|  |  |  |  |
| **14** | mir-30d | 56.31 | 66.39 |
|  | mir-30b | 520.55 | 637.33 |
|  |  |  |  |
| **15** | MiR-222 | 100.02 | 162.85 |
|  | MiR-221 | 3094.32 | 4661.52 |
|  |  |  |  |
| **16** | Mir-374b | 23.42 | 35.94 |
|  | Mir-421 | 9.59181 | 5.89777 |
|  |  |  |  |
| **17** | Mir-379 | 27.12 | 36.9 |
|  | Mir-411 | 951.37 | 848.32 |
|  | Mir-299 | 37.95 | 44.13 |
|  | Mir-1197 | 1.09 | 3.04 |
|  | Mir-323a | 6.16 | 12.93 |
|  | Mir-758 | 17.26 | 16.93 |
|  | Mir-494 | 0.41 | 0.76 |
|  | Mir-543 | 1.5 | 1.71 |
|  | Mir-495 | 3.83 | 3.8 |
|  | Mir-376c | 26.17 | 63.35 |
|  | Mir-654 | 19.45 | 17.11 |
|  | Mir-376b | 1.91 | 2.28 |
|  | Mir-1185-1 | 3.97 | 3.04 |
|  | Mir-1185-2 | 0.68 | 0.95 |
|  | Mir-381 | 2771.35 | 2215.66 |
|  | Mir-487b | 8.35858 | 7.99053 |
|  | Mir-539 | 11.36 | 9.51 |
|  | Mir-889 | 111.676 | 98.9304 |
|  | Mir-655 | 5.89211 | 15.2201 |
|  | Mir-487a | 3.28862 | 3.61476 |
|  | Mir-382 | 13.83 | 24.34 |
|  | Mir-134 | 25.3498 | 40.1429 |
|  | Mir-485 | 17.39 | 17.3 |
|  | Mir-154 | 9.86 | 22.06 |
|  | Mir-496 | 0.411077 | 0.190251 |
|  | Mir-377 | 33.15 | 29.28 |
|  | Mir-409 | 393.52 | 490.08 |
|  | Mir-369 | 16.44 | 20.92 |
|  | Mir-410 | 137.437 | 146.493 |
|  | Mir-656 | 4.11077 | 5.51727 |
|  |  |  |  |
| **18** | Mir-17 | 391.06 | 238.18 |
|  | Mir-18a | 41.92 | 45.84 |
|  | Mir-19a | 129.489 | 163.235 |
|  | Mir-20a | 791.45 | 374.79 |
|  | Mir-19b-1 | 1.91836 | 2.09276 |
|  | Mir-92a-1 | 18.9096 | 11.9858 |
|  |  |  |  |
| **19** | Mir-424 | 37.68 | 25.49 |
|  | Mir-503 | 1.22 | 1.71 |
|  | Mir-542 | 7.12 | 15.02 |
|  | Mir-450b | 5.61806 | 5.32702 |
|  |  |  |  |
| **20** | Mir-891b | 0.548103 | 0.951254 |
|  | Mir-892b | 0.137026 | 1.52201 |
|  | Mir-892a | 3.15159 | 3.80501 |
|  | Mir-888 | 1.09621 | 2.85376 |
|  | Mir-892c | 1.09621 | 1.71226 |
|  |  |  |  |
| **21** | Mir-371a | 0.274052 | 0.190251 |
|  | Mir-372 | 0.274052 | 0.380501 |
|  | Mir-373 | 2.05539 | 2.66351 |
|  |  |  |  |
| **22** | Mir-23b | 23.1574 | 132.034 |
|  | Mir-27b | 2627.87 | 3606.01 |
|  | Mir-3074 | 0.274052 | 0.190251 |
|  | Mir-24-1 | 0.411077 | 0.190251 |
|  |  |  |  |
| **23** | Mir-3677 | 1.91836 | 2.09276 |
|  | Mir-940 | 0.548103 | 1.1415 |
|  |  |  |  |
| **24** | Mir-23a | 708.96 | 1086.51 |
|  | Mir-27a | 882.99 | 1253.56 |
|  | Mir-24-2 | 0.274052 | 0.951254 |
|  |  |  |  |
| **25** | let-7f-1 | 0.137026 | 0.190251 |
|  | let-7d | 307.75 | 267.86 |
|  |  |  |  |
| **26** | Mir-100 | 11016.87 | 9493.89 |
|  | let-7a-2 | 18.3615 | 13.6981 |
|  |  |  |  |
| **27** | Mir-200c | 3.42565 | 4.75627 |
|  | Mir-141 | 0.685129 | 0.570752 |
|  |  |  |  |
| **28** | Mir-342 | 1.64431 | 6.08802 |
|  | Mir-151b | 8.63263 | 6.08802 |
|  |  |  |  |
| **29** | Mir-193b | 89.6 | 143.44 |
|  | Mir-365a | 15.2 | 15.79 |
|  |  |  |  |
| **30** | Mir-15b | 331.86 | 289.17 |
|  | Mir-16-2 | 4.52185 | 2.09276 |
|  |  |  |  |
| **31** | Mir-29b-1 | 25.07 | 7.03 |
|  | Mir-29a | 3685.58 | 3264.5 |
|  |  |  |  |
| **32** | miR-489 | 4.93293 | 2.66351 |
|  | miR-653 | 0.274052 | 0.190251 |

**Table 2: miRNA cluster analysis of the known miRNAs in normoxic and hypoxic U87MG cells**

In miR-493/136 cluster, all the miRNAs showed less than 300 TPM except miR-127 that is expressed 10 times greater (>3000 TPM) **(Figure 5a)**. Similarly, miR-379/656 cluster with 30 members showed a huge variation in the expression ranging from <0.5 TPM (miR-496) to >2000 TPM (miR-381) **(Figure 5b)**, while miR-17/92 cluster shows variation from ~2 (miR-19b) to >300 (miR-17) **(Figure 5c)**.

**Figure 5: MiRNA cluster analyses. Graphs representing expression of miRNAs belonging to clusters (a) miR-493/136 cluster (b) miR-379/656 cluster (c) miR-17/92 cluster is shown. A large variation in expression levels of different miRNAs present within the same cluster is observed in normoxic and hypoxic U87MG cells.**
