# Supplementary material for: Hypoxic signature of microRNAs in glioblastoma: insights from small RNA deep sequencing
Source: BMC Genomics. 2014 Aug 17;15(1):686. doi: 10.1186/1471-2164-15-686 (PMC4148931; doi:10.1186/1471-2164-15-686)
Supplement: Supplementary file 6 — Additional file 6: List of primers used for detection of specific miRNAs and mRNAs with Quantitative RT-PCR and for cloning wild type/mutated 3′ UTR of HIF3A. (DOCX 18 KB) [file 12864_2014_6378_MOESM6_ESM.docx]

|  | **List of primers used** |
| --- | --- |
|  |  |
| **Primer detail** | **Primer sequence** |
| **Primers for detection of mature microRNA level** | |
|  |  |
| **MIR-210-3p Forward Primer** | ATGCCTGTGCGTGTGAC |
| **MIR-1275 Forward Primer** | TATATGCGTGGGGGAGAG |
| **MIR-376c-3p Forward Primer** | CGCGCAACATAGAGGAA |
| **MIR-23b-3p Forward Primer** | ACGATCACATTGCCAGG |
| **MIR-193a-3p Forward Primer** | TGCAACTGGCCTACAAAG |
| **MIR-145-5p Forward Primer** | GAGTCCAGTTTTCCCAGG |
| **MIR-92b-3p Forward Primer** | CGTATTGCACTCGTCCC |
| **MIR-20a-5p Forward Primer** | GCGCTAAAGTGCTTATAGTGC |
| **MIR-10b-5p Forward Primer** | GCGTACCCTGTAGAACCG |
| **MIR-181a-2-3p Forward Primer** | GGACCACTGACCGTTGAC |
| **MIR-185-5p Forward Primer** | GCATGGAGAGAAAGGCAG |
| **iithsa_40**  **Forward Primer** | GCGATACAAGTCAGGCTCT |
| **iithsa_15**  **Forward Primer** | GCACCACAGGGTAGAACC |
| **iithsa_92**  **Forward Primer** | GCACAAATCTCCGGACC |
| **iithsa_1 Forward Primer** | CGCATGAGTACGGGATC |
| **iithsa_76 Forward Primer** | GCCTCATCCTGTCGCTA |
| **RNU6B**  **Forward Primer** | GCCCCTGCGCAAGGATGAC |
| **MIR-210-3p RT Primer** | GTCGTATCCAGTGCAGGGTCCGAGGTATTCGCACTGGATACGACTCAGCC |
| **MIR-1275 RT Primer** | GTCGTATCCAGTGCAGGGTCCGAGGTATTCGCACTGGATACGACGACAGC |
| **MIR-376c-3p RT Primer** | GTCGTATCCAGTGCAGGGTCCGAGGTATTCGCACTGGATACGACACGTGG |
| **MIR-23b-3p RT Primer** | GTCGTATCCAGTGCAGGGTCCGAGGTATTCGCACTGGATACGACGGTAAT |
| **MIR-193a-3p RT Primer** | GTCGTATCCAGTGCAGGGTCCGAGGTATTCGCACTGGATACGACACTGGG |
| **MIR-145-5p RT Primer** | GTCGTATCCAGTGCAGGGTCCGAGGTATTCGCACTGGATACGACAGGGAT |
| **MIR-92b-3p RT Primer** | GTCGTATCCAGTGCAGGGTCCGAGGTATTCGCACTGGATACGACGGAGGC |
| **MIR-20a-5p RT Primer** | GTCGTATCCAGTGCAGGGTCCGAGGTATTCGCACTGGATACGACCTACCT |
| **MIR-10b-5p RT Primer** | GTCGTATCCAGTGCAGGGTCCGAGGTATTCGCACTGGATACGACCACAAA |
| **MIR-181a-2-3p RT Primer** | GTCGTATCCAGTGCAGGGTCCGAGGTATTCGCACTGGATACGACGGTACAG |
| **MIR-185-5p RT Primer** | GTCGTATCCAGTGCAGGGTCCGAGGTATTCGCACTGGATACGACTCAGGA |
| **iithsa_40 RT Primer** | GTCGTATCCAGTGCAGGGTCCGAGGTATTCGCACTGGATACGACTAGGTC |
| **iithsa_15 RT Primer** | GTCGTATCCAGTGCAGGGTCCGAGGTATTCGCACTGGATACGACTGTCCG |
| **iithsa_92 RT Primer** | GTCGTATCCAGTGCAGGGTCCGAGGTATTCGCACTGGATACGACACTAAG |
| **iithsa_1 RT Primer** | GTCGTATCCAGTGCAGGGTCCGAGGTATTCGCACTGGATACGACTCTACA |
| **iithsa_76 RT Primer** | GTCGTATCCAGTGCAGGGTCCGAGGTATTCGCACTGGATACGACTCAGTG |
| **RNU6B RT**  **Primer** | GTCGTATCCAGTGCAGGGTCCGAGGTATTCGCACTGGATACGACAAAATATGGAAC |
| **Universal Reverse Primer** | GTGCAGGGTCCGAGGT |
| **Primers for detection of gene transcripts level and 3’ UTR cloning** | |
|  |  |
| **GAPDH Forward Primer** | GGTCGGAGTCAACGGATTTGGTCG |
| **GAPDH**  **Reverse Primer** | CTTCCAGGAGCGAGATCCCTC |
| **VEGF Forward Primer** | GCTACTGCCATCCAATCGAGAC |
| **VEGF Reverse Primer** | CTATGTGCTGGCCTTGGTGAG |
| **CA9 Forward Primer** | ACCTGGTGACTCTCGGCTACAG |
| **CA9 Reverse Primer** | CAGCCAGGCAGGAATTCAGC |
| **HIF1A Forward**  **Primer** | ACAGCAGCCAGACGATCATGC |
| **HIF1A Reverse**  **Primer** | CACAATCATAACTGGTCAGCTGTGG |
| **HIF3A Forward**  **Primer** | CATATTGCCAGGTGGGGAG |
| **HIF3A Reverse**  **Primer** | AGAGCGAGCAGTGCCTTC |
| **HIF3A 3’ UTR ForwardPrimer** | ATTACTAGTCCATCTGCCTTCTCCTCC |
| **HIF3A 3’ UTR Reverse Primer** | ATTACGCGTCCAAGAGAGGCAGAGAAAGG |
| **HIF3A mut 3’ UTR ForwardPrimer** | ACGCCGGCAGCCAACGGTGAGGATGGGGGCGCCAG |
| **HIF3A mut 3’ UTR Reverse primer** | CTGGCGCCCCCATCCTCACCGTTGGCTGCCGGCGT |
