# Supplementary material for: Hypoxic signature of microRNAs in glioblastoma: insights from small RNA deep sequencing
Source: BMC Genomics. 2014 Aug 17;15(1):686. doi: 10.1186/1471-2164-15-686 (PMC4148931; doi:10.1186/1471-2164-15-686)
Supplement: Supplementary file 8 — Additional file 8: List of HREs predicted in the promoter of miRNAs up-regulated in response to hypoxia, determined by deep sequencing and microarray profiling. The prediction was done using the program PROMO. (DOCX 15 KB) [file 12864_2014_6378_MOESM8_ESM.docx]

**List of HREs predicted in the promoter of miRNAs**

**miR-23b**

-- Factors predicted by PROMO in this sequence ----------------------
NAME; MATRIX_WIDTH;
HIF-1 [T01609]; 9

-- PROMO predictions detail ------------------------------------------

Sequence name; Factor name; Start position; End position; Dissimilarity; String; RE equally; RE query
Sequence; HIF-1 [T01609]; 923; 931; 2.554459; ACGTGCAAG; 0.11666; 0.12090;

**miR-132**

-- Factors predicted by PROMO in this sequence ----------------------
NAME; MATRIX_WIDTH;
HIF-1 [T01609]; 9

-- PROMO predictions detail ------------------------------------------

Sequence name; Factor name; Start position; End position; Dissimilarity; String; RE equally; RE query
Sequence; HIF-1 [T01609]; 3568; 3576; 0.906247; GCAGCACGT; 0.25296; 0.39867;

**miR-143**

-- Factors predicted by PROMO in this sequence ----------------------
NAME; MATRIX_WIDTH;
HIF-1 [T01609]; 9

-- PROMO predictions detail ------------------------------------------

Sequence name; Factor name; Start position; End position; Dissimilarity; String; RE equally; RE query
Sequence; HIF-1 [T01609]; 2017; 2025; 0.543748; GCTGCACGT; 0.11687; 0.11777;

**miR-145**

-- Factors predicted by PROMO in this sequence ----------------------
NAME; MATRIX_WIDTH;
HIF-1 [T01609]; 9

-- PROMO predictions detail ------------------------------------------

Sequence name; Factor name; Start position; End position; Dissimilarity; String; RE equally; RE query
Sequence; HIF-1 [T01609]; 289; 297; 0.543748; GCTGCACGT; 0.11646; 0.11282;
Sequence; HIF-1 [T01609]; 4180; 4188; 1.730353; CAGGCACGT; 0.23291; 0.22741;

**miR-152**

-- Factors predicted by PROMO in this sequence ----------------------
NAME; MATRIX_WIDTH;
HIF-1 [T01609]; 9

-- PROMO predictions detail ------------------------------------------

Sequence name; Factor name; Start position; End position; Dissimilarity; String; RE equally; RE query
Sequence; HIF-1 [T01609]; 3052; 3060; 2.735708; ACGTGCCAC; 0.11643; 0.11920;

**miR-154**

-- Factors predicted by PROMO in this sequence ----------------------
NAME; MATRIX_WIDTH;
HIF-1 [T01609]; 9

-- PROMO predictions detail ------------------------------------------

Sequence name; Factor name; Start position; End position; Dissimilarity; String; RE equally; RE query
Sequence; HIF-1 [T01609]; 102; 110; 2.008527; ACGTGCTCT; 0.32970; 0.25646;
Sequence; HIF-1 [T01609]; 4241; 4249; 0.834520; TCTGCACGT; 0.25212; 0.16442;

**miR-181d**

-- Factors predicted by PROMO in this sequence ----------------------
NAME; MATRIX_WIDTH;
HIF-1 [T01609]; 9

-- PROMO predictions detail ------------------------------------------

Sequence name; Factor name; Start position; End position; Dissimilarity; String; RE equally; RE query
Sequence; HIF-1 [T01609]; 4993; 5001; 2.021125; TGGGCACGT; 0.33313; 0.37644;

**miR-193a**

-- Factors predicted by PROMO in this sequence ----------------------
NAME; MATRIX_WIDTH;
HIF-1 [T01609]; 9

-- PROMO predictions detail ------------------------------------------

Sequence name; Factor name; Start position; End position; Dissimilarity; String; RE equally; RE query
Sequence; HIF-1 [T01609]; 165; 173; 0.834520; ACGTGCAGA; 0.25232; 0.32715;
Sequence; HIF-1 [T01609]; 4275; 4283; 0.906247; CCAGCACGT; 0.25232; 0.32715;

**miR-210**

-- Factors predicted by PROMO in this sequence ----------------------
NAME; MATRIX_WIDTH;
HIF-1 [T01609]; 9

-- PROMO predictions detail ------------------------------------------

Sequence name; Factor name; Start position; End position; Dissimilarity; String; RE equally; RE query
Sequence; HIF-1 [T01609]; 530; 538; 0.834520; TCTGCACGT; 0.25341; 0.39304;
Sequence; HIF-1 [T01609]; 873; 881; 1.005355; GGCGCACGT; 0.25341; 0.39304;
Sequence; HIF-1 [T01609]; 3924; 3932; 2.010710; ACGTGCGAG; 0.33138; 0.37456;

**miR-323a**

-- Factors predicted by PROMO in this sequence ----------------------
NAME; MATRIX_WIDTH;
HIF-1 [T01609]; 9

-- PROMO predictions detail ------------------------------------------

Sequence name; Factor name; Start position; End position; Dissimilarity; String; RE equally; RE query
Sequence; HIF-1 [T01609]; 2172; 2180; 2.554459; GTTGCACGT; 0.11641; 0.11801;

**miR-324**

-- Factors predicted by PROMO in this sequence ----------------------
NAME; MATRIX_WIDTH;
HIF-1 [T01609]; 9

-- PROMO predictions detail ------------------------------------------

Sequence name; Factor name; Start position; End position; Dissimilarity; String; RE equally; RE query
Sequence; HIF-1 [T01609]; 5033; 5041; 2.554459; ACGTGCAAG; 0.11634; 0.13088;

**miR-340**

-- Factors predicted by PROMO in this sequence ----------------------
NAME; MATRIX_WIDTH;
HIF-1 [T01609]; 9

-- PROMO predictions detail ------------------------------------------

Sequence name; Factor name; Start position; End position; Dissimilarity; String; RE equally; RE query
Sequence; HIF-1 [T01609]; 5006; 5014; 1.911603; GAAGCACGT; 0.33041; 0.28015;

**miR-374b**

-- Factors predicted by PROMO in this sequence ----------------------
NAME; MATRIX_WIDTH;
HIF-1 [T01609]; 9

-- PROMO predictions detail ------------------------------------------

Sequence name; Factor name; Start position; End position; Dissimilarity; String; RE equally; RE query
Sequence; HIF-1 [T01609]; 158; 166; 0.906247; ACGTGCTGG; 0.25153; 0.11894;

**miR-376c**

-- Factors predicted by PROMO in this sequence ----------------------
NAME; MATRIX_WIDTH;
HIF-1 [T01609]; 9

-- PROMO predictions detail ------------------------------------------

Sequence name; Factor name; Start position; End position; Dissimilarity; String; RE equally; RE query
Sequence; HIF-1 [T01609]; 994; 1002; 2.735708; ACGTGCCAC; 0.11595; 0.11275;
Sequence; HIF-1 [T01609]; 2981; 2989; 2.008527; ACGTGCTTT; 0.32853; 0.31635;

**miR-432**

-- Factors predicted by PROMO in this sequence ----------------------
NAME; MATRIX_WIDTH;
HIF-1 [T01609]; 9

-- PROMO predictions detail ------------------------------------------

Sequence name; Factor name; Start position; End position; Dissimilarity; String; RE equally; RE query
Sequence; HIF-1 [T01609]; 1510; 1518; 0.543748; CCTGCACGT; 0.11659; 0.18102;
Sequence; HIF-1 [T01609]; 1635; 1643; 0.543748; ACGTGCAGC; 0.11659; 0.18102;

**miR-450a**

-- Factors predicted by PROMO in this sequence ----------------------
NAME; MATRIX_WIDTH;
HIF-1 [T01609]; 9

-- PROMO predictions detail ------------------------------------------

Sequence name; Factor name; Start position; End position; Dissimilarity; String; RE equally; RE query
Sequence; HIF-1 [T01609]; 3822; 3830; 1.549104; ACGTGCACG; 0.23305; 0.16208;

**miR-454**

-- Factors predicted by PROMO in this sequence ----------------------
NAME; MATRIX_WIDTH;
HIF-1 [T01609]; 9

-- PROMO predictions detail ------------------------------------------

Sequence name; Factor name; Start position; End position; Dissimilarity; String; RE equally; RE query
Sequence; HIF-1 [T01609]; 4011; 4019; 2.832632; ATGGCACGT; 0.13659; 0.12694;

**miR-542**

-- Factors predicted by PROMO in this sequence ----------------------
NAME; MATRIX_WIDTH;
HIF-1 [T01609]; 9

-- PROMO predictions detail ------------------------------------------

Sequence name; Factor name; Start position; End position; Dissimilarity; String; RE equally; RE query
Sequence; HIF-1 [T01609]; 2822; 2830; 1.549104; ACGTGCACG; 0.23332; 0.21545;

**miR-574**

-- Factors predicted by PROMO in this sequence ----------------------
NAME; MATRIX_WIDTH;
HIF-1 [T01609]; 9

-- PROMO predictions detail ------------------------------------------

Sequence name; Factor name; Start position; End position; Dissimilarity; String; RE equally; RE query
Sequence; HIF-1 [T01609]; 1641; 1649; 1.646028; ACGTGCATT; 0.23328; 0.17802;

**miR-598**

-- Factors predicted by PROMO in this sequence ----------------------
NAME; MATRIX_WIDTH;
HIF-1 [T01609]; 9

-- PROMO predictions detail ------------------------------------------

Sequence name; Factor name; Start position; End position; Dissimilarity; String; RE equally; RE query
Sequence; HIF-1 [T01609]; 4566; 4574; 1.646028; AGTGCACGT; 0.23332; 0.23188;

**miR-655**

-- Factors predicted by PROMO in this sequence ----------------------
NAME; MATRIX_WIDTH;
HIF-1 [T01609]; 9

-- PROMO predictions detail ------------------------------------------

Sequence name; Factor name; Start position; End position; Dissimilarity; String; RE equally; RE query
Sequence; HIF-1 [T01609]; 4326; 4334; 2.832632; ACGTGCCAT; 0.13610; 0.13140;

**miR-664a**

-- Factors predicted by PROMO in this sequence ----------------------
NAME; MATRIX_WIDTH;
HIF-1 [T01609]; 9

-- PROMO predictions detail ------------------------------------------

Sequence name; Factor name; Start position; End position; Dissimilarity; String; RE equally; RE query
Sequence; HIF-1 [T01609]; 1998; 2006; 1.646028; AATGCACGT; 0.23264; 0.10781;

**miR-1275**

-- Factors predicted by PROMO in this sequence ----------------------
NAME; MATRIX_WIDTH;
HIF-1 [T01609]; 9

-- PROMO predictions detail ------------------------------------------

Sequence name; Factor name; Start position; End position; Dissimilarity; String; RE equally; RE query
Sequence; HIF-1 [T01609]; 633; 641; 2.021125; ACGTGCCCA; 0.32944; 0.33165;

**miR-1307**

-- Factors predicted by PROMO in this sequence ----------------------
NAME; MATRIX_WIDTH;
HIF-1 [T01609]; 9

-- PROMO predictions detail ------------------------------------------

Sequence name; Factor name; Start position; End position; Dissimilarity; String; RE equally; RE query
Sequence; HIF-1 [T01609]; 2111; 2119; 0.543748; ACGTGCAGG; 0.11785; 0.09525;

**miR-29b**

-- Factors predicted by PROMO in this sequence ----------------------
NAME; MATRIX_WIDTH;
HIF-1 [T01609]; 9

-- PROMO predictions detail ------------------------------------------

Sequence name; Factor name; Start position; End position; Dissimilarity; String; RE equally; RE query
Sequence; HIF-1 [T01609]; 1558; 1566; 1.730353; ACGTGCCTG; 0.23259; 0.14753;

**miR-149**

-- Factors predicted by PROMO in this sequence ----------------------
NAME; MATRIX_WIDTH;
HIF-1 [T01609]; 9

-- PROMO predictions detail ------------------------------------------

Sequence name; Factor name; Start position; End position; Dissimilarity; String; RE equally; RE query
Sequence; HIF-1 [T01609]; 3373; 3381; 0.724998; ACGTGCCGG; 0.11648; 0.25068;

**miR-494**

-- Factors predicted by PROMO in this sequence ----------------------
NAME; MATRIX_WIDTH;
HIF-1 [T01609]; 9

-- PROMO predictions detail ------------------------------------------

Sequence name; Factor name; Start position; End position; Dissimilarity; String; RE equally; RE query
Sequence; HIF-1 [T01609]; 1377; 1385; 1.197019; TCAGCACGT; 0.25197; 0.26023;
Sequence; HIF-1 [T01609]; 1382; 1390; 1.730353; ACGTGCCTG; 0.23259; 0.23740;

**miR-877**

-- Factors predicted by PROMO in this sequence ----------------------
NAME; MATRIX_WIDTH;
HIF-1 [T01609]; 9

-- PROMO predictions detail ------------------------------------------

Sequence name; Factor name; Start position; End position; Dissimilarity; String; RE equally; RE query
Sequence; HIF-1 [T01609]; 266; 274; 1.730353; CAGGCACGT; 0.23282; 0.21665;
Sequence; HIF-1 [T01609]; 271; 279; 2.735708; ACGTGCCAC; 0.11641; 0.11052;
Sequence; HIF-1 [T01609]; 2212; 2220; 1.730353; CAGGCACGT; 0.23282; 0.21665;
Sequence; HIF-1 [T01609]; 2217; 2225; 2.735708; ACGTGCCAC; 0.11641; 0.11052;

**miR-1225**

-- Factors predicted by PROMO in this sequence ----------------------
NAME; MATRIX_WIDTH;
HIF-1 [T01609]; 9

-- PROMO predictions detail ------------------------------------------

Sequence name; Factor name; Start position; End position; Dissimilarity; String; RE equally; RE query
Sequence; HIF-1 [T01609]; 1659; 1667; 0.543748; CCTGCACGT; 0.11650; 0.23211;
Sequence; HIF-1 [T01609]; 1950; 1958; 1.911603; CGAGCACGT; 0.33009; 0.34767;
Sequence; HIF-1 [T01609]; 2360; 2368; 0.906247; GCAGCACGT; 0.25242; 0.40716;
Sequence; HIF-1 [T01609]; 3439; 3447; 1.102279; ACGTGCGCT; 0.25242; 0.40716;

**miR-1915**

-- Factors predicted by PROMO in this sequence ----------------------
NAME; MATRIX_WIDTH;
HIF-1 [T01609]; 9

-- PROMO predictions detail ------------------------------------------

Sequence name; Factor name; Start position; End position; Dissimilarity; String; RE equally; RE query
Sequence; HIF-1 [T01609]; 591; 599; 1.730353; ACGTGCCCC; 0.23254; 0.24304;
