# Supplementary material for: Hypoxic signature of microRNAs in glioblastoma: insights from small RNA deep sequencing
Source: BMC Genomics. 2014 Aug 17;15(1):686. doi: 10.1186/1471-2164-15-686 (PMC4148931; doi:10.1186/1471-2164-15-686)
Supplement: Supplementary file 9 — Additional file 9: Quantitative RT-PCR data showing miRNA levels in response to hypoxia. Graph showing miRNAs upregulated (a) or downregulated (b) in response to hypoxia in U251MG cells. The graphical data points represent mean ± S.D. of at least three independent experiments. (*P > 0.01 and < 0.05; **P < 0.01). Error bars denote ± S. D. (PPTX 73 KB) [file 12864_2014_6378_MOESM9_ESM.pptx]

## Slide 1
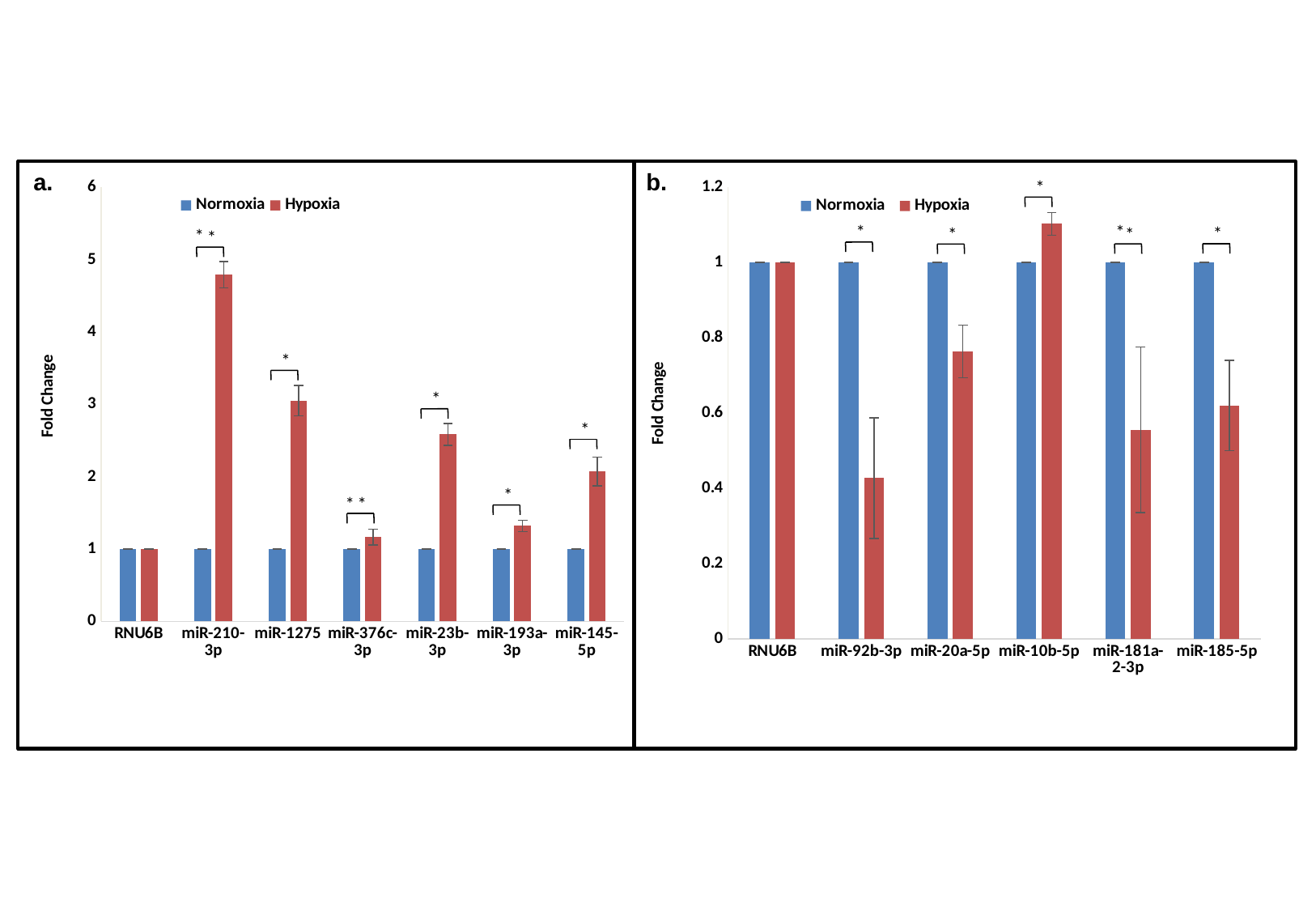

a.
b.
*
### Chart
| Category | Normoxia | Hypoxia |
|---|---|---|
| RNU6B | 1.0 | 1.0 |
| miR-210-3p | 1.0 | 4.789914818475718 |
| miR-1275 | 1.0 | 3.052518417921117 |
| miR-376c-3p | 1.0 | 1.164733586468455 |
| miR-23b-3p | 1.0 | 2.58470566127498 |
| miR-193a-3p | 1.0 | 1.3195079107728929 |
| miR-145-5p | 1.0 | 2.070529847682757 |
### Chart
| Category | Normoxia | Hypoxia |
|---|---|---|
| RNU6B | 1.0 | 1.0 |
| miR-92b-3p | 1.0 | 0.42631744588397835 |
| miR-20a-5p | 1.0 | 0.7631296044802794 |
| miR-10b-5p | 1.0 | 1.1019051158766084 |
| miR-181a-2-3p | 1.0 | 0.5547847360339234 |
| miR-185-5p | 1.0 | 0.6198538499694929 |*
*
*
*
*
*
*
*
*
*
*
*
*
