# Supplementary material for: Hypoxic signature of microRNAs in glioblastoma: insights from small RNA deep sequencing
Source: BMC Genomics. 2014 Aug 17;15(1):686. doi: 10.1186/1471-2164-15-686 (PMC4148931; doi:10.1186/1471-2164-15-686)
Supplement: Supplementary file 11 — Additional file 11: Quantitative RT-PCR data showing the miR-210-3p level in a miR-210-3p overexpressing U87MG cell line or control U87MG cells (a) and U87MG cells transiently transfected with miR-210-3p inhibitor or control oligos (b), respectively. The graphical data points represent mean ± S.D. of at least three independent experiments. (*P > 0.01 and < 0.05; **P < 0.01). Error bars denote ± S. D. (PPTX 60 KB) [file 12864_2014_6378_MOESM11_ESM.pptx]

## Slide 1
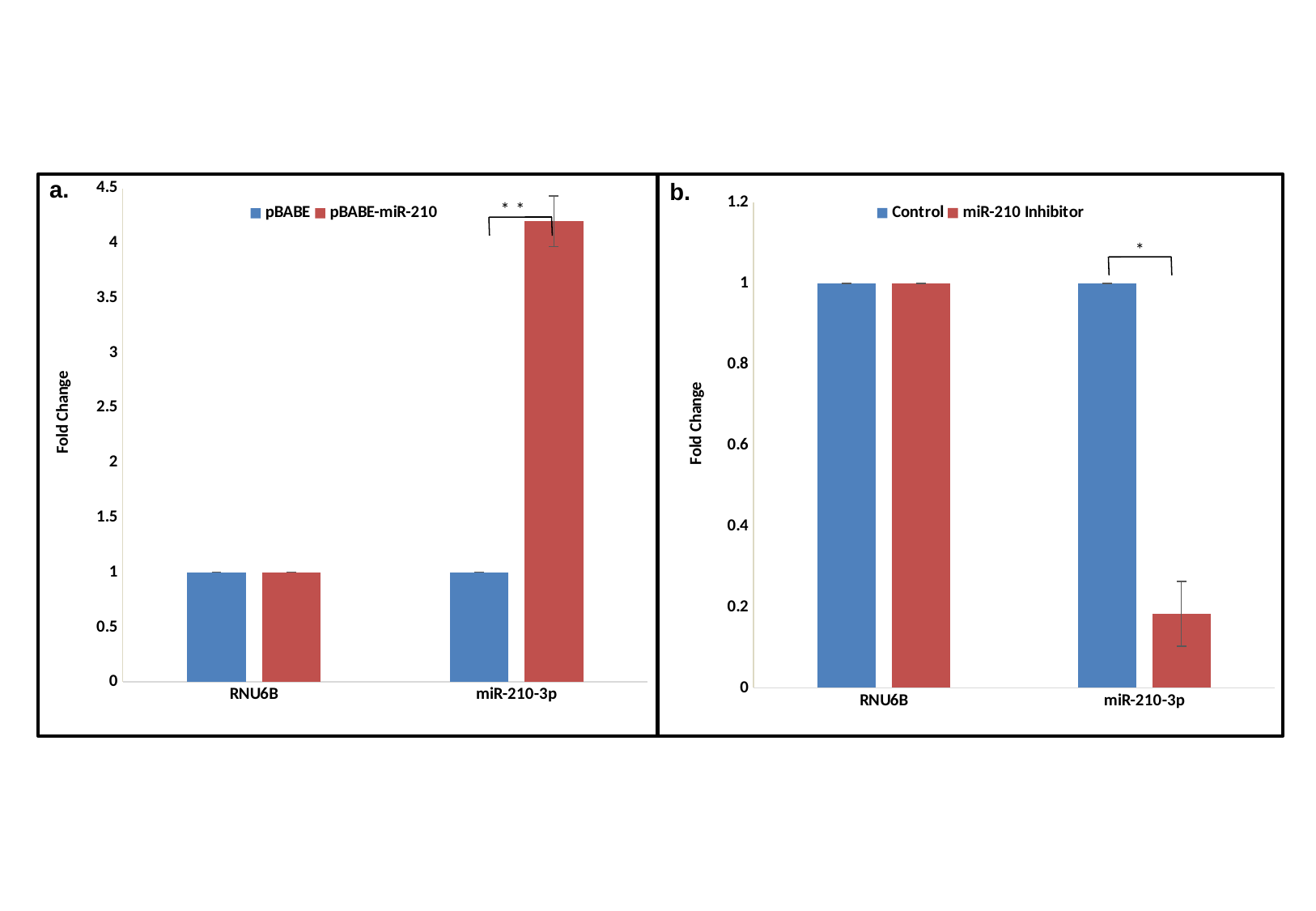

a.
b.
### Chart
| Category | pBABE | pBABE-miR-210 |
|---|---|---|
| RNU6B | 1.0 | 1.0 |
| miR-210-3p | 1.0 | 4.19886673449228 |
### Chart
| Category | Control | miR-210 Inhibitor |
|---|---|---|
| RNU6B | 1.0 | 1.0 |
| miR-210-3p | 1.0 | 0.18301071199320323 |*
*
*
